# Differentiating Nontuberculous Mycobacterial Pulmonary Disease from Pulmonary Tuberculosis in Resource-Limited Settings: A Pragmatic Model for Reducing Misguided Antitubercular Treatment

**DOI:** 10.3390/healthcare13091065

**Published:** 2025-05-05

**Authors:** Wei Zhang, Jun Chen, Zhenhua Chen, Jun Quan, Zebing Huang

**Affiliations:** 1 Hunan Key Laboratory of Viral Hepatitis, Department of Infectious Diseases, Xiangya Hospital Central South University, Changsha 410008, China; csuxyzw@csu.edu.cn (W.Z.); drcjun@csu.edu.cn (J.C.); 2 Hunan Institute for Tuberculosis Control, Hunan Chest Hospital, Changsha 410013, China; drzhchen@foxmail.com; 3 National Clinical Research Center for Geriatric Disorders, Xiangya Hospital Central South University, Changsha 410008, China

**Keywords:** nontuberculous mycobacterial pulmonary disease, pulmonary tuberculosis, differential diagnosis, bronchiectasis, clinical prediction model, resource-limited settings, multivariable logistic regression, receiver operating characteristic curve

## Abstract

**Background**: Differentiating nontuberculous mycobacterial pulmonary disease (NTM-PD) from pulmonary tuberculosis (PTB) remains challenging due to overlapping clinical features, particularly in resource-limited settings where diagnostic errors are frequent. This retrospective case–control study (January 2023–June 2024) aimed to identify key clinical predictors and develop a diagnostic model to distinguish NTM-PD from PTB. **Methods**: Patients initially presumed to have PTB (meeting clinical–radiological criteria but lacking bacteriological confirmation at admission) at a tertiary tuberculosis hospital were enrolled. Final diagnoses of NTM-PD (*n* = 105) and PTB (*n* = 105) were confirmed by mycobacterial culture identification. Clinical, laboratory, and radiological data were compared using univariate analysis. Variables showing significant differences (*p* < 0.05) were entered into multivariable logistic regression. Diagnostic performance was evaluated via receiver operating characteristic (ROC) curve analysis. **Results**: Female sex (odds ratio [OR] = 2.51, 95% confidence interval [CI] 1.12–5.60), hemoptysis (OR = 2.20, 1.05–4.62), bronchiectasis (OR = 5.92, 2.56–13.71), and emphysema/pulmonary bullae (OR = 2.69, 1.16–6.24) emerged as independent predictors of NTM-PD, while systemic symptoms favored PTB (OR = 0.45, 0.20–0.99). The model demonstrated 91.4% specificity and 68.6% sensitivity with an area under the curve [AUC] of 0.871. **Conclusions**: This high-specificity model helps prioritize NTM-PD confirmation in females with hemoptysis and structural lung changes (computed tomography evidence of bronchiectasis and/or emphysema) while maintaining PTB suspicion when systemic symptoms (fever, night sweats, weight loss) dominate. The approach may reduce misguided antitubercular therapy in resource-limited settings awaiting culture results.

## 1. Introduction

Pulmonary diseases caused by mycobacteria, particularly *Mycobacterium tuberculosis* (MTB) and nontuberculous mycobacteria (NTM), pose significant global health challenges. Pulmonary tuberculosis (PTB), caused by MTB, remains a leading cause of infectious disease-related mortality, with an estimated 10 million new cases and 1.3 million deaths annually worldwide [1]. In contrast, nontuberculous mycobacterial pulmonary disease (NTM-PD) has emerged as a growing concern, especially in aging populations and individuals with structural lung diseases [2]. While PTB incidence has declined in many regions, NTM-PD prevalence has risen sharply. In China, for instance, the prevalence of NTM-PD reached 6.8% (458/6766) among sputum smear-positive tuberculosis-suspected patients in 2021 [3], with a nationwide NTM detection rate of 6.4% (317/4917 isolates) reported in the same year [4], paralleling trends observed in high-income countries such as the United States (47.48 per 100,000 in individuals >65 years) [5]. NTM-PD now represents an emerging respiratory disease burden worldwide, demonstrating consistent upward epidemiological trajectories in both developed and developing nations [6,7].

The rising incidence of NTM-PD is multifactorial. Environmental factors, including widespread water disinfection favoring NTM survival [8], and host susceptibility linked to chronic lung diseases [9], immunosuppressive therapies [10], and antibiotic overuse [11], have been implicated. Improved diagnostic techniques, such as nucleic acid amplification tests and matrix-assisted laser desorption/ionization time-of-flight mass spectrometry, have also enhanced detection [12,13]. However, differentiating NTM-PD from PTB remains challenging. Both diseases share clinical features (e.g., cough, weight loss) and radiological findings (e.g., cavitations, nodules) [14]. Conventional acid-fast staining cannot distinguish MTB from NTM, while antigenic cross-reactivity confounds serological tests [15]. Although mycobacterial culture is the diagnostic gold standard, its utility is limited by prolonged turnaround times (4–8 weeks) [16]. Misdiagnosis carries grave consequences. PTB requires standardized antitubercular therapy (ATT), whereas NTM-PD demands prolonged multidrug regimens (e.g., macrolides, aminoglycosides) with higher toxicity and cost [17]. Inappropriate ATT for NTM-PD exacerbates drug resistance and delays effective treatment. Despite these risks, no validated clinical algorithm exists to guide early differentiation, particularly in patients initially presumed to have PTB—a critical gap this study addresses. While previous work by Kendall et al. [18] identified age, birthplace, and chronic obstructive pulmonary disease (COPD) as discriminators between TB and NTM disease, their findings were primarily derived from low-TB-incidence regions (e.g., the United States) with distinct demographic profiles (predominantly White populations), limiting generalizability to high-TB-burden, resource-limited settings like China. Moreover, their model relied on basic demographic and symptomatic features, omitting detailed clinical–laboratory–radiological discriminators (e.g., inflammatory markers, immune profiles, and structural lung disease characteristics such as bronchiectasis).

To address above critical gaps, we conducted this retrospective study with two primary objectives: (1) to identify key clinical, radiological, and laboratory predictors differentiating NTM-PD from PTB in a TB-endemic setting and (2) to develop a pragmatic diagnostic model optimized for resource-limited clinical workflows. By analyzing comprehensive profiles of 210 presumptive PTB patients (105 NTM-PD vs. 105 PTB), this study aims to reduce misguided antitubercular therapy through evidence-based differentiation prior to bacteriological confirmation.

## 2. Materials and Methods

### 2.1. Patients

A total of 150 patients initially presumed to have PTB upon admission (meeting predefined criteria: clinical—chronic cough ≥2 weeks with ≥1 TB-associated symptom/sign [hemoptysis, fever, night sweats, weight loss, abnormal auscultation, or lymphadenopathy]; radiographic—active TB-like lesions assessed independently by two board-certified radiologists) were retrospectively screened. All cases were ultimately confirmed as NTM-PD through mycobacterial culture and speciation at the Tuberculosis Hospital between January 2023 and June 2024. After applying exclusion criteria, 105 NTM-PD patients remained eligible for inclusion in the study. Additionally, 105 patients ultimately confirmed as having PTB during the same period were randomly selected from the pool of admitted PTB patients.

All selected patients were initially diagnosed as having suspected PTB at the time of admission. Patients with NTM-PD were diagnosed according to the ATS/ERS/ESCMID/IDSA clinical practice guideline [19], and patients with PTB were diagnosed based on the “Diagnosis of Pulmonary Tuberculosis” (WS288-2017) issued by the National Health Commission of the People’s Republic of China [20]. The exclusion criteria were as follows: (1) both NTM and MTB being positive according to bacterial culture; (2) extrapulmonary NTM-related diseases; (3) extrapulmonary tuberculosis; (4) immunodeficiency diseases, such as advanced cancer or HIV; and (5) patients with missing data. This study was approved by the hospital’s medical ethics review board (approval No. LS2022101401). Written informed consent was obtained from all participants with data anonymization (ID codes replacing personal identifiers). Confidentiality was maintained through encrypted electronic records and password-protected databases accessible only to the research team.

### 2.2. Collection of Clinical Data

General Information: Data on age, gender, height, body weight, symptoms and the time from symptom onset to diagnosis were collected for each patient.

Laboratory Tests: The laboratory tests included blood routine examination and serum total protein (TP), albumin (ALB), globulin (GLB), immunoglobulins (IgA, IgG, and IgM), complements (C3 and C4), C-reactive protein (CRP), procalcitonin (PCT), erythrocyte sedimentation rate (ESR), tubercle bacillus antibody, T-cell Spot of Interferon-gamma Release Assay (T-SPOT), and purified protein derivative (PPD) skin tests, as well as sputum acid-fast staining and mycobacterial cultures.

Imaging Examinations: Computed tomography (CT) scans were performed to evaluate lung involvement and detect structural abnormalities. All CT images were independently reviewed by two experienced radiologists, and any discrepancies were resolved through discussion to ensure standardized and reliable assessment.

Comorbidities: The presence of comorbidities, including COPD and diabetes mellitus (DM), was documented.

Previous Medical History (PMH): Data on previous medical history were collected, including information on history of chest trauma or surgery, and chronic dust exposure.

### 2.3. Statistical Analysis

All data were analyzed using SPSS 25.0 software. Continuous variables were assessed for normality using Shapiro–Wilk tests and equality of variances using Levene’s tests. Variables conforming to normal distribution were expressed as mean ± standard deviation (X ± SD) and compared using independent t-tests, while non-normally distributed variables were presented as median (interquartile range) [M(IQR)] with Mann–Whitney U tests. Categorical variables were expressed as frequencies (percentages) and analyzed using Pearson’s χ^2^ tests or Fisher’s exact tests when expected cell counts were <5. Variables showing statistical significance (*p* < 0.05) were entered into multivariable backward stepwise logistic regression. A receiver operating characteristic (ROC) curve was constructed to evaluate the diagnostic performance of the regression model with internal validation. A *p*-value < 0.05 was considered statistically significant and all reported *p*-values are two-sided.

## 3. Results

### 3.1. Demographic and Clinical Characteristics of NTM-PD and PTB Patients

**Figure 1:**
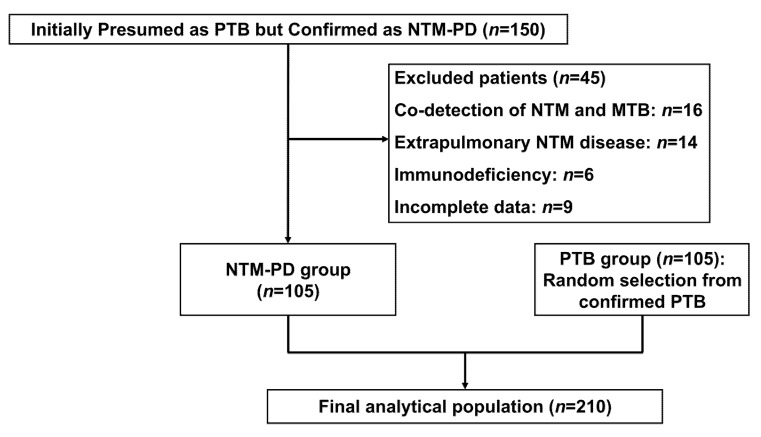
Enrollment flowchart for the case–control study of NTM-PD versus PTB patients (January 2023–June 2024).

Between January 2023 and June 2024, 150 patients initially presumed to have PTB but ultimately confirmed as NTM-PD were screened. After excluding 45 cases due to co-detection of NTM/MTB (*n* = 16), extrapulmonary NTM diseases (*n* = 14), immunodeficiency (*n* = 6), or incomplete data (*n* = 9), 105 NTM-PD patients were included. A case–control of 105 PTB patients was randomly selected from admitted patients during the same period, yielding a final study population of 210 cases (Figure 1).

Our comparative analysis revealed distinct demographic, clinical, and radiological patterns between NTM-PD and PTB patients across three key domains: baseline characteristics, laboratory/microbiological profiles, and thoracic imaging findings. Initial comparisons of baseline characteristics (Table 1) demonstrated significant intergroup differences. NTM-PD patients were older (53.1 ± 14.3 vs. 45.1 ± 17.6 years, *p* < 0.05) and more likely to be female (59.0% vs. 36.2%, *p* < 0.05) compared to PTB patients. The NTM-PD group also had lower mean body mass index (BMI) (19.19 ± 3.36 vs. 20.59 ± 3.13 kg/m^2^, *p* < 0.05) and median symptom-to-diagnosis interval: 16 (IQR 5–37) vs. 3 (IQR 2–9) months, *p* < 0.05.

Comorbidity patterns diverged markedly: structural lung diseases predominated in NTM-PD, with higher rates of COPD (12.4% vs. 4.8%, *p* < 0.05). Conversely, metabolic disorders like DM were more prevalent in PTB (17.1% vs. 4.8%, *p* < 0.05). A history of thoracic trauma/surgery was uniquely associated with NTM-PD (7.6% vs. 1.0%, *p* < 0.05).

Clinically, NTM-PD patients more frequently presented with hemoptysis (51.4% vs. 27.6%, *p* < 0.05) and dyspnea (72.4% vs. 55.2%, *p* < 0.05), whereas PTB patients showed heightened systemic manifestations (59.0% vs. 40.0%, *p* < 0.05), particularly night sweats (28.6% vs. 16.2%, *p* < 0.05) and weight loss (32.4% vs. 16.2%, *p* < 0.05).

### 3.2. Laboratory and Microbiological Characteristics

Beyond demographic disparities, laboratory parameters revealed pathophysiological distinctions. As shown in Table 2, NTM-PD patients’ routine blood analysis exhibited significantly lower neutrophil counts (3.53 vs. 4.23 × 10^9^/L, *p* < 0.05), monocyte counts (0.47 vs. 0.51 × 10^9^/L, *p* < 0.05), monocyte-to-lymphocyte ratios (0.30 vs. 0.45, *p* < 0.05), and platelet counts (211 vs. 251 × 10^9^/L, *p* < 0.05) compared to PTB patients. Serum protein analysis further showed reduced globulin levels in NTM-PD (26.3 vs. 29.4 g/L, *p* < 0.05), though albumin levels were comparable between groups.

Immunological testing highlighted complement system differences: NTM-PD patients had lower C3 (1.15 vs. 1.26 g/L, *p* < 0.05) and C4 (0.27 vs. 0.31 g/L, *p* < 0.05) levels, whereas immunoglobulin (IgG/IgA/IgM) levels showed no intergroup differences. Inflammatory markers were markedly elevated in PTB, with higher CRP (22.4 vs. 5.6 mg/L, *p* < 0.05) and ESR (34 vs. 23 mm/h, *p* < 0.05).

Microbiological profiling demonstrated critical diagnostic divergences. PTB patients showed higher rates of strong mycobacterial culture positivity (3+/4+: 84.7% vs. 43.8%) and immunodiagnostic positivity (T-SPOT: 80.0% vs. 41.9%; TB antibody: 35.2% vs. 17.1%, both *p* < 0.05). PPD reactivity differed substantially, with 57.1% of NTM-PD patients testing negative versus 19.0% in PTB. Sputum acid-fast staining positivity rates were comparable (46.7% vs. 42.9%).

### 3.3. Comparative Analysis of Thoracic CT Features

Radiological evaluation further corroborated these clinical differences. CT imaging revealed distinct patterns between NTM-PD and PTB patients (Table 3). Bilateral lung involvement was more prevalent in NTM-PD (90.5% vs. 76.2%, *p* < 0.05), with preferential right middle lobe and left lingular lobe involvement (77.1% vs. 56.2%, *p* < 0.05). Chronic structural changes predominated in NTM-PD, evidenced by higher rates of fibrous stripes (73.3% vs. 51.4%, *p* < 0.05), bronchiectasis (61.9% vs. 15.2%, *p* < 0.05), and emphysema/bullae (45.7% vs. 21.0%, *p* < 0.05). In contrast, acute inflammatory manifestations such as consolidation were more frequent in PTB (21.0% vs. 8.6%, *p* < 0.05). Cavitation showed comparable prevalence (47.6% vs. 53.3%, *p* < 0.05).

Notably, CT findings demonstrate 4.1-fold-higher bronchiectasis prevalence in NTM-PD, establishing it as the strongest radiological predictor. The bilateral/multilobar involvement (90.5%/93.3%) reflects NTM-PD’s chronic disseminated nature.

### 3.4. Multivariable Predictors and Diagnostic Model Performance

**Figure 2:**
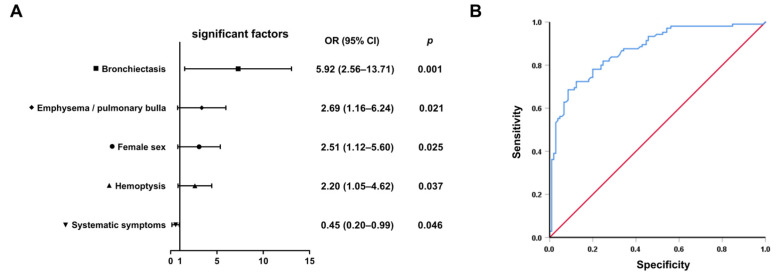
Development and validation of the differential diagnostic model. (**A**) Forest plot of adjusted odds ratios with 95% confidence intervals from the final multivariable logistic regression model (*n* = 210). (**B**) ROC curve demonstrating the model’s discriminative capacity. Blue stepped curve: proposed prediction model (AUC = 0.871 [95% CI: 0.823–0.919]); red diagonal line: reference line of random guessing (AUC = 0.50).

Variables showing significant differences in univariate analyses (*p* < 0.05, Table 1, Table 2 and Table 3) were entered into backward stepwise logistic regression. The final model identified five independent discriminators (Table 4): female gender (OR = 2.51, 95% CI: 1.12–5.60, *p* = 0.025), bronchiectasis (OR = 5.92, 95% CI: 2.56–13.71, *p* = 0.001), hemoptysis (OR = 2.20, 95% CI: 1.05–4.62, *p* = 0.037), emphysema/bullae (OR = 2.69, 95% CI: 1.16–6.24, *p* = 0.021), and systemic symptoms (OR = 0.45, 95% CI: 0.20–0.99, *p* = 0.046). Notably, bronchiectasis demonstrated the strongest association with NTM-PD, increasing the odds by nearly sixfold. Systemic symptoms including fever, night sweats, and weight loss favored PTB diagnosis, consistent with MTB’s greater systemic inflammatory potential (Figure 2A).

The composite model demonstrated discriminative accuracy with an AUC of 0.871 (95% CI 0.823–0.919) on receiver operating characteristic (ROC) analysis (Figure 2B). At the optimal cutoff value, specificity reached 91.4% (95% CI 84.6–95.5) with 68.6% sensitivity (95% CI 58.9–77.0), indicating strong potential for ruling-in NTM-PD diagnosis while minimizing false-positive identification; the 91.4% specificity ensures <9% false-positive rate when prioritizing NTM-PD confirmation. The model’s high specificity holds particular clinical relevance given the critical need to avoid unnecessary antitubercular therapy in patients ultimately diagnosed with NTM-PD. This diagnostic characteristic is particularly valuable in resource-limited clinical scenarios.

## 4. Discussion

Our study establishes a pragmatic diagnostic model demonstrating high specificity (91.4%) for distinguishing NTM-PD from PTB in resource-limited settings. The model identifies four independent predictors of NTM-PD—female sex, hemoptysis, bronchiectasis, and emphysema/pulmonary bullae—while systemic symptoms favor PTB. These findings align with but critically extend prior epidemiological and pathophysiological insights. Female predisposition aligns with national surveillance data showing elevated NTM-PD risk in women (adjusted OR = 1.8), potentially reflecting hormonal influences on immune responses [3].The robust association between bronchiectasis and NTM-PD (OR = 5.92) corroborates multinational evidence identifying structural lung damage as the strongest risk factor (meta-OR = 21.4), likely through impaired mucociliary clearance and persistent mycobacterial colonization [3,9]. Notably, the prolonged symptom-to-diagnosis interval in NTM-PD (median 16 vs. 3 months in PTB) reflects both diagnostic complacency due to AFB smear positivity and the indolent nature of NTM infections, often leading to delayed recognition of chronic colonization. The strong association between bronchiectasis and NTM-PD (OR = 5.92) underscores the bidirectional relationship between structural lung damage and NTM colonization, where impaired mucociliary clearance facilitates mycobacterial persistence, while chronic NTM infection exacerbates airway remodeling [21,22,23].

This mechanistic synergy is further evidenced by the higher prevalence of prior thoracic trauma/surgery in NTM-PD (18.1% vs. 6.9%), suggesting iatrogenic structural alterations may create niches for NTM persistence—a phenomenon documented in post-lobectomy patients [24,25]. Notably, 61.9% of NTM-PD patients exhibited radiologically confirmed bronchiectasis, far exceeding rates in PTB (15.2%), suggesting this feature could serve as a cornerstone for early suspicion of NTM-PD in tuberculosis-endemic regions. The female predominance in NTM-PD (59.0% vs. 36.2% in PTB) aligns with global epidemiological trends but remains mechanistically incompletely understood [5,26]. The lower BMI in NTM-PD patients (20.3 vs. 22.1 kg/m^2^) may reflect chronic nutritional depletion from prolonged infection, though established evidence identifies low BMI as an independent predisposing factor for NTM-PD [27]. Existing evidence indicates that estrogen may modulate macrophage-mediated mycobacterial clearance [28], with postmenopausal women demonstrating increased susceptibility to NTM infections [29]. This biological plausibility is further supported by the absence of gender disparity in PTB cohorts, where MTB’s virulence likely overrides sex-specific immune differences [30]. Clinically, this emphasizes the need for heightened NTM suspicion in older women presenting with chronic respiratory symptoms, particularly when structural lung abnormalities are present.

The distinct symptom profiles between groups offer actionable diagnostic clues. While hemoptysis was more frequent in NTM-PD (51.4% vs. 27.6%), PTB patients exhibited pronounced systemic inflammation (59.0% vs. 40.0%), reflected in elevated CRP (22.4 vs. 5.6 mg/L) and ESR (34 vs. 23 mm/h). This inflammatory divergence is also mirrored in hematological parameters: the lower monocyte-to-lymphocyte ratio (MLR) in NTM-PD (0.28 vs. 0.43) contrasts with its established role as a diagnostic biomarker in active tuberculosis [31]. This dichotomy likely reflects pathogen-specific immune modulation: MTB triggers robust Th1/Th17-mediated responses driving fever and weight loss [32,33], whereas NTM’s indolent course favors localized rather than systemic manifestations [34,35]. Such divergence underscores the clinical value of symptom stratification when microbiological confirmation is pending. Notably, the higher prevalence of DM in PTB patients (17.1% vs. 4.8%) aligns with known mechanisms by which hyperglycemia impairs innate and adaptive immunity, favoring MTB progression. Although DM was not retained as an independent predictor in the final model, its potential confounding effects were statistically adjusted. Future studies should explore how DM-associated metabolic dysregulation differentially modulates host responses to MTB versus NTM.

The model’s high specificity (91.4%) addresses a critical gap in tuberculosis triage systems prevalent across resource-constrained settings. In clinical workflows where molecular diagnostics remain inaccessible, clinicians could integrate this prediction model at two pivotal decision points: (1) during initial evaluation of smear-positive patients, where ≥2 predictors (e.g., female with hemoptysis and CT-confirmed bronchiectasis) should trigger immediate mycobacterial culture speciation rather than empirical ATT initiation, and (2) when managing culture-negative TB suspects, prioritizing NTM-PD investigations for patients exhibiting the identified predictors. This approach aligns with the WHO’s tuberculosis strategy in high-TB-burden regions [36,37,38] by reducing unnecessary ATT prescriptions while awaiting confirmatory results—particularly crucial given the 16-month median diagnostic delay observed in NTM-PD cases. Implementation could follow a stepped protocol: radiographic identification of bronchiectasis/emphysema prompts targeted symptom assessment (hemoptysis frequency, absence of systemic symptoms), with female patients automatically flagged for expedited NTM testing. Such integration would optimize existing resources, as CT imaging and basic symptom screening are widely available even in peripheral clinics. In contrast, night sweats with weight loss should maintain PTB suspicion regardless of structural abnormalities, ensuring ATT continuity for true TB cases. This dual-pathway model operationalizes predictive features into actionable clinical logic, bridging the diagnostic gap between sputum smear positivity and delayed culture results.

While this study provides clinically actionable insights, several limitations merit consideration. First, the retrospective case–control design introduces potential selection bias, particularly regarding control group selection from a tertiary TB hospital population, which may not fully represent community-level diagnostic challenges. Second, single-center recruitment from a high-TB-incidence region limits generalizability to settings with differing NTM species distributions or healthcare infrastructures. Third, the exclusion of immunocompromised populations precludes extrapolation to these high-risk groups where NTM-PD pathophysiology and radiological patterns may diverge substantially. Fourth, the absence of NTM speciation data prevents analysis of species-specific clinical-radiological correlations, which could refine predictive accuracy given varying virulence across NTM species. Furthermore, the inclusion of nonspecific inflammatory markers and immune parameters may introduce diagnostic ambiguity, as these biomarkers could be elevated in concurrent infections or autoimmune conditions. While their inclusion reflects real-world diagnostic workflows in resource-limited settings, this underscores the need for future studies to validate our model against populations with diverse inflammatory comorbidities. Finally, treatment outcome data were not analyzed, leaving unanswered whether early model-guided NTM-PD suspicion improves therapeutic success rates compared to standard diagnostic pathways.

Future research should prioritize multicenter prospective validation across diverse epidemiological contexts, incorporation of immunocompromised cohorts, and cost-effectiveness analyses of model-implemented diagnostic algorithms versus empirical ATT approaches. Integration of rapid molecular diagnostics with our clinical model could further reduce diagnostic delays. Longitudinal studies assessing the model’s impact on antimicrobial stewardship metrics would strengthen implementation evidence.

## 5. Conclusions

Our study identifies female sex, hemoptysis, bronchiectasis, and emphysema/pulmonary bullae as key predictors of NTM-PD, while systemic symptoms favor PTB. These findings advocate for a paradigm shift in presumptive PTB management in resource-limited settings. Clinicians should prioritize NTM-PD confirmation via sputum culture or NAATs in patients with ≥2 predictors, even if acid-fast bacilli are detected and maintain PTB as the primary diagnosis when systemic symptoms (fever, night sweats, weight loss) predominate, regardless of structural lung changes. This dual-strategy approach balances diagnostic urgency with antimicrobial stewardship, particularly where mycobacterial speciation is delayed. Future implementation studies should validate this model’s impact on reducing misguided ATT.

## Figures and Tables

**Table 1:** Demographic and clinical characteristics of NTM-PD and PTB patients.

Variables	NTM-PD	PTB	*p*
Age (years)	53.1 ± 14.3	45.1 ± 17.6	0.001
Female [*n* (%)]	62 (59.0)	38 (36.2)	0.001
Height (cm)	161.40 ± 7.27	163.56 ± 7.95	0.041
Weight (kg)	50.68 ± 9.39	53.56 ± 10.46	0.037
BMI (kg/m^2^)	19.19 ± 3.36	20.59 ± 3.13	0.009
Time from symptom onset to diagnosis (month)	16 (5~37)	3 (2~9)	0.001
Comorbidities [*n* (%)]			
COPD	13 (12.4)	5 (4.8)	0.049
DM	5 (4.8)	18 (17.1)	0.004
PMH [*n* (%)]			
Chest trauma and surgery	8 (7.6)	1 (1.0)	0.041
Chronic dust exposure	6 (5.7)	6 (5.7)	1.000
Symptoms [*n* (%)]			
Expectoration	85 (81.0)	77 (73.3)	0.189
Irritating dry cough	6 (5.7)	11 (10.5)	0.206
Hemoptysis	54 (51.4)	29 (27.6)	0.001
Dyspnea	76 (72.4)	58 (55.2)	0.010
Chest pain	14 (13.3)	10 (9.5)	0.386
Systemic symptoms ^1^	42 (40.0)	62 (59.0)	0.006
Fever	14 (13.3)	24 (22.9)	0.073
Night sweats	17 (16.2)	30 (28.6)	0.031
asthenia	10 (9.5)	9 (8.6)	0.810
Weight loss	17 (16.2)	34 (32.4)	0.006

^1^ Systemic symptoms include fever, night sweats, asthenia, and weight loss. Continuous variables with normal distribution are presented as mean ± standard deviation (SD); non-normally distributed variables are expressed as median [interquartile range] (IQR). Categorical variables are expressed as counts (percentages).

**Table 2:** Laboratory parameter comparison between NTM-PD and PTB patients.

Test Items	NTM-PD	PTB	*p*
Routine blood test			
WBC (×10^9^/L)	5.92 (4.88~7.55)	6.68 (5.01~8.42)	0.144
Neutrophils (×10^9^/L)	3.53 (2.78~4.94)	4.23 (3.02~6.00)	0.020
Lymphocytes (×10^9^/L)	1.43 (1.10~1.91)	1.35 (0.93~1.66)	0.059
Monocytes (×10^9^/L)	0.47 (0.35~0.63)	0.51 (0.42~0.76)	0.042
ML ratio ^1^	0.30 (0.21~0.55)	0.45 (0.29~0.64)	0.001
PLT (×10^9^/L)	211 (163~275)	251 (202~341)	0.001
Serum proteins (g/L)			
TP	66.4 (62.6~71.2)	69.8 (63.5~74.9)	0.010
ALB	39.6 (36.8~43.0)	40.2 (34.6~43.7)	0.601
GLB	26.3 (23.7~28.9)	29.4 (26.4~32.7)	0.001
Immunological test			
C3 (g/L)	1.15 (1.01~1.27)	1.26 (1.17~1.49)	0.001
C4 (g/L)	0.27 (0.23~0.31)	0.31 (0.26~0.36)	0.004
IgG (g/L)	14.8 (12.2~16.6)	15.4 (12.4~18.2)	0.276
IgA (g/L)	2.67 (1.96~3.65)	2.49 (1.84~3.17)	0.234
IgM (g/L)	1.28 (0.80~1.79)	1.07 (0.73~1.68)	0.197
Infection indicators			
CRP (mg/L)	5.6 (1.4~33.2)	22.4 (6.9~49.1)	0.001
PCT (ng/mL)	0.16 (0.11~0.32)	0.19 (0.13~0.41)	0.312
ESR (mm/h)	23 (11~47)	34 (15~69)	0.013
PPD test [*n* (%)]			0.005
Negative	60 (57.1)	20 (19.0)	
Weakly positive	6 (5.7)	10 (9.5)	
Positive	25 (23.8)	52 (49.5)	
Strongly positive	14 (13.3)	23 (21.9)	
TB antibody [*n* (%)]			
Positive	18 (17.1)	37 (35.2)	0.003
T-SPOT [*n* (%)]			
Positive	44 (41.9)	84 (80.0)	0.001
Sputum acid-fast stain ^2^ [*n* (%)]			0.280
+	17 (16.2)	12 (11.4)	
++	18 (17.1)	11 (10.5)	
+++	9 (8.6)	10 (9.5)	
++++	5 (4.8)	11 (10.5)	
Sputum culture ^3^ [*n* (%)]		50	0.001
+	23 (21.9)	7 (6.7)	
++	36 (34.3)	9 (8.6)	
+++	38 (36.2)	25 (23.8)	
++++	8 (7.6)	64 (60.9)	
1+~2+:3+~4+ ratio	59/46	16/89	0.001

^1^ Monocyte-to-lymphocyte ratio; ^2^ acid-fast staining grading (acid-fast bacilli [AFB] per microscopic field): (1+) 1–9 AFB/100 fields; (2+) 1–9 AFB/10 fields; (3+) 1–9 AFB/field; (4+) >10 AFB/field; ^3^ mycobacterial culture semi-quantitation grading: (1+) colonies covering ≤25% of culture area; (2+) colonies covering >25%~50% of culture area; (3+) colonies covering >50%~75% of culture area; (4+) colonies covering >75%~100% of culture area. Continuous variables with normal distribution are presented as mean ± standard deviation (SD); non-normally distributed variables are expressed as median [interquartile range] (IQR). Categorical variables are expressed as counts (percentages).

**Table 3:** Comparative analysis of thoracic CT features between NTM-PD and PTB.

CT Findings [*n* (%)]	NTM-PD	PTB	*p*
Bilateral lung lesions	95 (90.5)	80 (76.2)	0.005
Multiple lobe involvement	98 (93.3)	89 (84.8)	0.047
Upper lobe	89 (84.8)	78 (74.3)	0.060
Right middle lobe/Left lingual lobe	81 (77.1)	59 (56.2)	0.001
Lower lobe	83 (79.0)	87 (82.9)	0.482
Patchy shadow	89 (84.8)	84 (80.0)	0.365
Fibrous stripe	77 (73.3)	54 (51.4)	0.001
Nodular shadow	73 (69.5)	76 (72.4)	0.648
Ground glass opacity	4 (3.8)	7 (6.7)	0.353
Consolidation	9 (8.6)	22 (21.0)	0.011
Cavity	50 (47.6)	56 (53.3)	0.408
Bronchiectasis	65 (61.9)	16 (15.2)	0.001
Emphysema/pulmonary bulla	48 (45.7)	22 (21.0)	0.001
Atelectasis	19 (18.1)	11 (10.5)	0.115
Pleural effusion	16 (15.2)	24 (22.9)	0.160

Continuous variables with normal distribution are presented as mean ± standard deviation (SD); non-normally distributed variables are expressed as median [interquartile range] (IQR). Categorical variables are expressed as counts (percentages).

**Table 4:** Final multivariable model for differentiating NTM-PD from PTB.

Risk Factors	β Regression Coefficient	*p*	OR	95%CI
NTM-PD predictors				
Female sex	0.919	0.025	2.51	1.12–5.60
Bronchiectasis	1.779	0.001	5.92	2.56–13.71
Hemoptysis	0.789	0.037	2.20	1.05–4.62
Emphysema/pulmonary bulla	0.991	0.021	2.69	1.16–6.24
PTB predictor				
Systemic symptoms ^1^	−0.802	0.046	0.45	0.20–0.99

^1^ Systemic symptoms include fever, night sweats, asthenia, and weight loss.

## Data Availability

The datasets used and analyzed during the present study are available from the corresponding author upon reasonable request.
